# Challenges in Humoral Immune Response to Adeno-Associated Viruses Determination

**DOI:** 10.3390/ijms26020816

**Published:** 2025-01-19

**Authors:** Daria A. Naumova, Tatyana Krokunova, Denis Maksimov, Olga N. Mityaeva, Ekaterina A. Astakhova, Pavel Yu Volchkov

**Affiliations:** 1 Federal Research Center for Original and Prospective Biomedical and Pharmaceutical Technologies, 8 Baltiyskaya Street, Moscow 125315, Russia; 2 Moscow Center for Advanced Studies, Kulakova Street, 20, Moscow 123592, Russia

**Keywords:** AAV, neutralizing antibodies, antigenic cartography, anti-AAV antibodies, humanized models, NHP models, mice models

## Abstract

Adeno-associated viruses (AAVs) are non-pathogenic, replication-deficient viruses that have gained widespread attention for their application as gene therapy vectors. While these vectors offer high transduction efficiency and long-term gene expression, the host immune response poses a significant challenge to their clinical success. This review focuses on the obstacles to evaluating the humoral response to AAVs. We discuss the problems with the validation of in vitro tests and the possible approaches to overcome them. Using published data on neutralizing titers of AAV serotypes, we built the first antigenic maps of AAVs in order to visualize the antigenic relationships between varying serotypes.

## 1. Introduction

Adeno-associated viral vectors are thought to be a prospective delivery platform for gene therapies due to their high transduction and long-term expression efficiency in different cell types, non-pathogenic nature, and low immune responses in humans [1,2]. AAVs are not known to induce any human diseases [3]; however, they naturally circulate in certain host animals. People may be spontaneously infected by AAVs during life, which can lead to increased levels of AAV-specific serum antibodies [4,5]. As a result, up to 80% of the human population has neutralizing antibodies to AAVs [6], with humoral response to AAV2 being most prevalent [7]. T-cell-mediated immune responses to AAVs are rarely detected in treatment-naive individuals because of low-sensitivity tests or other reasons [8,9,10,11,12]. However, this review is focused on humoral responses to AAVs and methods of evaluating antibody titers to AAVs.

The pre-existing humoral immune response is thought to be a deleterious factor for AAV-based gene therapy because of the neutralization vector caused by specific serum antibodies [13,14,15]. Despite the fact that AAVs are used as a platform for vaccination against infectious agents [16] and cancers [17,18], the role of the pre-existing immune response to AAVs in such areas is less studied because the AAV doses used are significantly lower than in gene therapies. A high level of pre-existing anti-AAV antibodies may promote phagocytosis and complement activation [14]. Phagocytized vectors are not processed for transgene expression [19]. As a result, a higher dose of the drug is required to achieve therapeutic goals. According to the FDA (U.S. Food and Drug Administration), high titers of anti-AAV antibodies are an exclusion criterion in gene therapy clinical trials in which systemic administration is used [20].

Over 30 clinical studies of AAV-based gene therapy (GT) that reported anti-AAV serum antibody titers were analyzed in this study. We found that cutoff titer values varied from study to study, regardless of local or systemic vector administration, the method of measuring titers, or the serotype selected for GT delivery. However, in order to fairly compare cutoff titers from different clinical trials, antibody titer tests need to have been conducted in similar conditions. We found that the protocols of these assays are often not detailed enough in the descriptions of clinical trials or scientific papers. The differences in providing these assays have a direct influence on the titer values.

The correct determination of the anti-AAV titer is important not only for the definition of the exclusion criteria for GT but also when comparing humoral responses to different serotypes of AAVs. A serotype-switching approach using low cross-reactive wild-type AAVs or synthetic AAVs may be used to overcome a pre-existing humoral response [21]. In such cases, the direct comparison of sera antibody activity against serotypes of interest is paramount. ELISA (enzyme-linked immunosorbent assay) and in vitro neutralization assays with permissive cell lines are the most common methods used to determine the level of the anti-AAV antibody response [22]. Although ELISA is relatively easy to standardize, in vitro neutralization assays have limitations for AAVs. Here, we discuss the theoretical basement of the methodological problems of modern in vitro assays. In particular, we show why the ratio of full and empty capsids is important in in vitro neutralization assays, how the ratio of serum antibodies and used viral particles influences the result, and why the differences in the transduction efficiency of cell lines between AAV serotypes are a crucial problem with this assay [23,24]. Some recommendations to overcome these problems are proposed.

The other approach to determining the humoral response to AAVs is to use animal models with a humanized immune system. Regardless of the obvious difficulties in the usage of animals, there is a possibility for AAVs to transduce different cell types in the whole organism and interact with specific antibodies or immune cells [25]. In this paper, we have reviewed animal models that are used to study human-like humoral responses to AAVs with subsequent in vitro neutralization assays. Moreover, we describe an animal model approach that allows the complete avoidance of in vitro neutralization assays for the determination of human sera neutralization titers to AAVs.

Although animal models have several advantages compared to in vitro assays, the latter are more suitable for screening assays and are more easy to standardize [26]. The previously listed disadvantages of most current in vitro assays influence the level of transduced target cells, which are used to evaluate the neutralization titers. As a result, different neutralization titers reflect not only the optimized conditions of the assay but also the antigenic characteristics of different serotypes. However, if antibody titers to different AAV serotypes are obtained in similar conditions, their antigenic characteristics can be evaluated and visualized with various methods, such as antigenic cartography [27]. In the case of AAV serotypes, such maps may be useful in revealing the direction in which novel synthetic AAV synthesis should be taken to create AAVs that will antigenically differ from the wild type, to which pre-existing immunity is often present. As far as we know, no AAV antigenic maps have been created yet. Here, we constructed an AAV antigenic map using previously published neutralization titer data.

The present review discusses the pros and cons of current in vitro assays and animal models to detect human humoral immune responses to AAVs and proposes possible improvements. Overcoming the limitations of current test systems will allow for better analysis and comparison of data in order to overcome pre-existing humoral responses. This makes this review relevant to researchers and clinicians seeking to enhance the safety and efficacy of gene therapies.

## 2. Generation of Anti-AAV Abs 

AAVs transduce a wide spectrum of target cells, including antigen-presenting cells (APCs) [28,29]. In in vitro experiments, AAVs were uptaken predominantly by monocyte-derived dendritic cells (moDCs) and monocytes, conventional and plasmacytoid DCs, and neutrophils to a lesser degree [14]. AAV-proposed PAMPs, such as capsid proteins and CpG in viral genomes [30], interact with PRRs (TLR2 and endosomal TLR9, respectively) on or within the cells that trigger an inflammatory reaction and the maturation of dendritic cells, their migration to the lymph nodes [8], and their presentation to naive CD8^+^ or CD4^+^ T cells. Jamie L. Shirley et al. provided some insights into this process. They showed that activation of TLR9 on pDCs, in which it is highly expressed, led to type 1 interferon production that, in combination with CD40L costimulation, activated the maturation of conventional DCs (cDCs) and the presentation of capsid antigens in their MHC I [31]. After endocytosis by target cells or APCs, AAVs undergo proteasomal degradation following endosomal escape [32], capsid phosphorylation, and ubiquitination [31]. As a result, capsid antigens are presented onto MHC class I or cross-presented onto MHC II. Activated APCs trigger a specific T-cell response. Activated CD4^+^ T_FH_ cells induce B cell activation and antibody secretion. In addition, in in vitro and in vivo mouse models, the AAV capsid was shown to trigger IL-1β- and IL-6-dependent B cell differentiation and specific IgM and IgG secretion [33].

In fact, the process of humoral response to AAV induction is not well-studied. There are certain challenges with studying this process due to the absence of an acute inflammation stage after infection with AAVs in humans. Moreover, the study of the initiation of the immune response to AAVs requires high-quality purified vectors. For instance, Qingyun Zheng et al. have shown that rAAV-DJ vectors yielded from DH5α but not ClearColi K12 bacterial strains induced the inflammatory response in cell cultures in vitro and in C57BL/6 mice retinas in vivo [34]. This response was mediated by lipopolysaccharide contamination in the used AAV sample.

However, the presence of a humoral response to AAVs was repeatedly shown, and the level of this response is higher in adults than in children [5]. The level of pre-existing anti-AAV Abs is crucial in AAV-based gene therapies. For example, recently, it was proven in vitro that high titers of anti-AAV neutralizing antibodies (NAbs) trigger complement activation that may determine thrombotic microangiopathy [14].

## 3. Anti-AAV Antibody Titers as Exclusion Criteria in Clinical Trials

Pre-existing AAV-specific antibodies hamper the efficient transduction of AAVs and potentially lead to decreased delivery of the gene therapy candidate [6]. No correlation has been shown between anti-AAV Ab levels and adverse events in gene therapy (GT) studies [35]. However, this may be explained by the fact that, in at least 45 percent of U.S. Food and Drug Administration (FDA)-approved GTs with the systemic injection of AAVs, patients with pre-existing Abs to AAVs are often excluded from clinical trials [36]. The enrollment criteria have been set at either an anti-AAV NAb titer <1:10 or a total anti-AAV-IgG titer <1:100 [19].

The exclusion of patients from clinical trials with high levels of anti-AAV antibodies greatly depends on the therapeutic area. The deleterious impact of pre-existing antibodies on the therapeutic effect is expected to be more significant when the administration is systemic, as compared to local administration [36]. A meta-analysis by Hau Kiu Edna Au et. al. (2022) showed that almost 90% of blood disease studies exclude patients with pre-existing antibodies, while only <10% of eye disease and 21% of CNS disease studies exclude such patients [21]. However, FDA guidance recommends the exclusion of seropositive patients for both local and systemic administration [37,38,39,40].

Titer exclusion criteria differ between studies. In Table 1, we summarized these data from various clinical studies. Trials were extracted from the U.S National Library of Medicine database (ClinicalTrials.gov), the largest clinical trials database to date, following the keyword search for “AAV” and using a cutoff of 23 July 2021. Only studies with exclusion criteria for the presence of NAbs in AAVs were selected. It should be noted that these titers seemingly do not show absolute titers of the ADA (anti-drug antibody).

Table 1 shows heterogeneity in the approaches to anti-AAV determination: some studies use ELISA to measure binding antibodies, while others use neutralization tests with cell cultures to measure NAbs or transduction inhibition tests. Titer values vary from 1:2 to 1:20 for neutralizing antibody titers and from 1:50 to 1:1600 for binding antibody titers, which confirms the lack of uniform standards for this parameter. The insufficiently precise wording of the exclusion criteria in a number of clinical studies is also questionable.

In most of the clinical trials presented in Table 1, there is no information on how titers were defined. Apparently, it is a serum dilution that exceeds the cutoff value, which was also not defined. By the recommendation of the FDA, the cutoff level of the ADA should be determined using samples from treatment-naïve subjects [41]. Bioanalytical laboratories establish the cutoff for antibody assessment as being when 1–10% of the tested treatment-naive samples are false-positive [35].

The detection of binding or neutralizing antibodies before and after drug injection is required to understand the immunogenicity, safety, and efficacy of gene therapy products based on AAVs. It would be beneficial to have standard protocols in order to compare the data obtained from different studies on the immunogenicity of AAV vectors.

**Figure 1:**
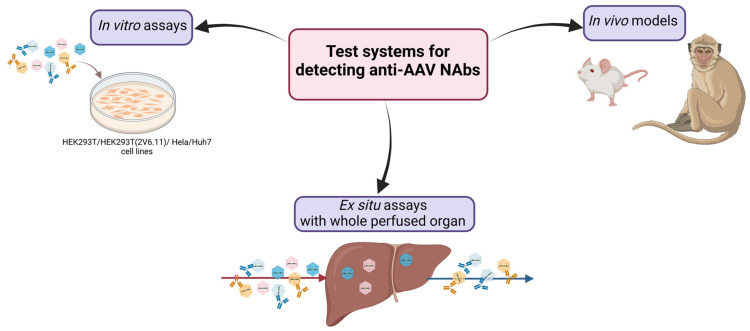
The present test systems for detecting anti-adeno-associated virus (AAV) neutralizing antibodies (NAbs).

As Table 1 shows, ELISA and in vitro neutralization tests are the most commonly used in clinical trials to determine anti-AAV Ab titers. Although some studies show that binding and neutralizing AAV-specific IgGs correlate well [11], measuring NAbs is thought to be more desirable. Currently used assays for detecting anti-AAV NAbs include in vitro assays, in vivo models, and ex situ assays (Figure 1). In vitro assays are suitable for screening assays due to their simplicity and speed. However, in scientific areas where more precise characteristics of a vector are required, in vivo models and ex situ assays may be used to obtain more information about the vector of interest.

We discuss below the advantages and disadvantages of the current assays used for evaluating serum NAbs to AAVs.

## 4. In Vitro Neutralization Assay

The NAb titer to AAVs is one of the most important criteria used for excluding patients for clinical trials and gene therapy treatment. Most clinical trials use in vitro assays to test NAb levels in patients’ sera, because they are easy to set up and give consistent results [42].

The choice of target cell line is an important factor that influences the results of the in vitro virus neutralization assay (VNA). For SARS coronaviruses, the key cell receptor (ACE-2) is well-characterized [43]. Since the beginning of the COVID-19 pandemic, it has been relatively quickly shown that ACE-2 is a key cell receptor for the novel SARS-CoV-2 virus [44]. It enabled the rapid development and validation of a good in vitro model to detect anti-SARS-CoV-2 NAbs. Engineered cells with stable expression of ACE-2 are target cells in this assay [45]. Then, the expression of TMPRSS was added to target cell lines [46], which are now widely used in scientific studies and clinical trials.

Unlike SARS-CoV-2, there are no well-defined key cell receptors that are responsible for AAV transduction to the cells. Thus, glycan receptors are suggested to be low-specificity attachment factors, but they are not ‘primary receptors’ [47]. The potential key role of AAVR has also been discussed [48]. Therefore, different cell lines are used in in vitro neutralization assays for AAVs. The HEK293, Hela, COS-7, GM16095, and Huh7 cell lines are most used to determine the level of anti-AAV NAbs in vitro today, although the transduction efficiencies of these cell lines vary by up to five orders of magnitude among different AAV serotypes [49,50]. The low transduction efficiency of several serotypes makes it impossible to determine NAb titers due to the limit of detection. A higher multiplicity of infections (MOIs) can be used to overcome this problem, but it leads to underestimating NAb titers [42]. Moreover, lower MOIs in terms of in vitro VNAs enable the cost of the experiment to be reduced because they require a lower number of AAVs. As a result, more permissive cell lines suitable for transduction by most serotypes are highly desirable.

To improve transduction efficiency, target cells may be infected with adenovirus 5 before the neutralization assay [4,51,52]. 2V6.11, a cell line obtained from HEK293, overexpresses the adenovirus gene, E4 ORF, under the control of the ecdysone-inducible promoter. It was shown [22] that AAV6, 8, and 9 transduce this cell line more effectively than Huh7, HEK293, and HeLa, respectively. Hoi Yee Chow et al. established a Hela cell line with stable overexpression of AAVR and showed the increased sensitivity of NAbs detection for different AAV serotypes [50]. Yi-lin Xie et al. used melittin peptide and have shown that the pre-incubation of rAAV2 with this peptide, but not insertion of melittin into the rAAV2 capsid, enhances vector transduction efficiency in HEK293 and Huh7 cells in vitro [53].

The choice of cell line for in vitro neutralization assays is not the only important choice. Typically, VNAs consist of two steps: incubating viral particles with diluted serum and adding this mixture to target cells. Choosing the optimal dose of the viral vector is pivotal when the goal is to compare neutralization titers against different serotypes. Three VNT methods and their limitations are described below. Ultimately, good principles have been proposed. These principles are applied to VNA in general, in which other viruses are used.

**Figure 2:**
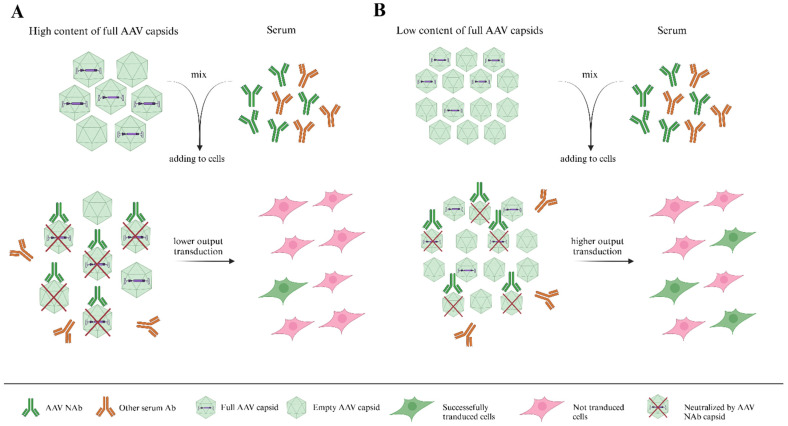
A figure illustrating the importance of considering the number of full and empty capsids in neutralization tests. Serotype A (subfigure (**A**)) and serotype B (subfigure (**B**)) take in equal numbers of viral particles, as measured by viral genomes. However, the serotype B sample contains a lower percentage of full capsids than serotype A. Adeno-associated virus (AAV) neutralizing antibodies (NAbs) bind not only full capsids but also empty capsids, so the number of cells transduced by serotype B is higher.

The first and most widespread approach is to use a similar number of viral particles (measured by viral genomes, for example, Figure 2). The first limitation of this approach is that it does not consider the number of full and empty capsids. Empty particles compete with full AAV particles for binding to serum NAbs [51] and reduce the number of NAbs, blocking functional (full) AAVs. As a result, a large quantity of full AAVs can transduce target cells and express the reporter transgene, which will influence titration curves and neutralization titers.

For this reason, we propose that quality control tests of virus particles (VPs) are required. Tests of the ratio of full/empty capsids and the presence of VP aggregates may be appropriate. Taking viruses with a similar ratio of full/empty capsids is a good practice.

**Figure 3:**
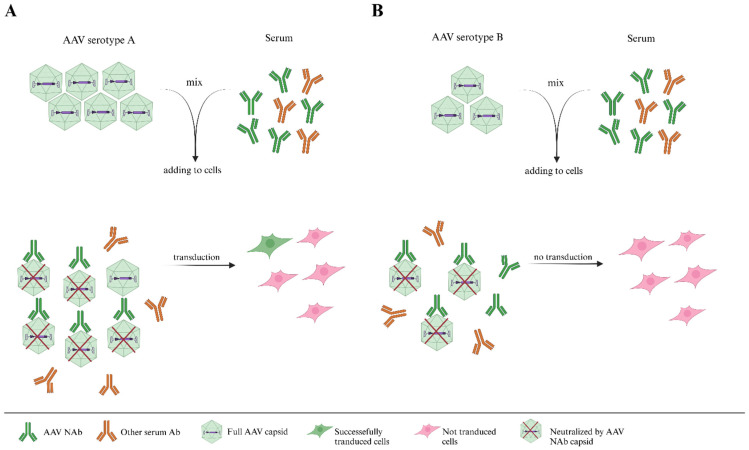
A figure illustrating the importance of the variance in the multiplicity of infections (MOIs) of adeno-associated virus (AAV) serotypes. Chosen doses of serotype A (subfigure (**A**)) and serotype B (subfigure (**B**)) transduce an equal number of target cells in the absence of serum antibodies. However, in the presence of an equal volume/number of specific AAV antibodies from serum, the levels of transduced target cells are different, which reflects the lower obtained viral dosage of serotype A, rather than the difference in antigenic characteristics between the two serotypes.

The second approach is to use the number of viral particles (VPs) that transduce a similar percentage of target cells [54] (Figure 3). This approach is limited by the varying ability of different serotypes to infect cells. Thus, in order to achieve the same level of transduction for different serotypes, it is necessary to select a number of VPs, which, in some cases, may differ by an order of magnitude. Chimeric AAVs are very important and are often purposefully constructed to improve the abilities of transduction target cells [55,56,57]. Sera samples are traditionally serially 2-fold diluted and mixed with the chosen viral dosage. However, if the chosen dosages of viruses are dramatically distinguished, the ratio of serum antibodies and viral particles will be dramatically different. The lower number of VPs will require a lower number of NAbs to be neutralized, which will also have an influence on neutralization titers against different serotypes.

Thus, our recommendation is to use an equal ratio between the number of VPs and the volume of serum samples. The limitation that has affected both of the above approaches is the differing transduction levels of AAV serotypes. In our opinion, this limitation is the most difficult to overcome. If an equal number of AAVs are used in an experiment for different serotypes, the transduction level may vary from 0 to 100% for different serotypes. Low transduction levels (about 10%) increase the significance of mistakes related to measurement, which will have an influence on titration curves and neutralization titers. If the number of transduced cells is close to 100%, it is not always clear what number of AAVs transduce cells in abundance. The appropriate transduction level for different serotypes may not always be achieved. Alternatively, if transduction level is used as a metric, then the same transduction level may be achieved by vastly different levels of VPs, with variances as high as several degrees of magnitude.

As previously discussed, incubation with an equal ratio of VPs and serum volume is needed. Thus, we suggest incubating VPs with serum and transferring the volume of this mixture, which, in the absence of serum, would transduce the necessary percentage of target cells. However, following this recommendation becomes challenging when MOIs differ by more than three orders of magnitude.

Unfortunately, in a number of articles on NAb titer assessment for AAVs, the methods used for assessment are not described in sufficient detail. Studies use different MOIs [4], different genomic MOIs [58,59], and different genomic numbers of AAVs [58] for different AAV serotypes.

## 5. Determination of the Immune Response to AAVs in Animal Models

As discussed above, the most difficult limitation to overcome in an in vitro assay is the different transduction efficiency of cell lines. It should be underlined that this limitation is only relevant when the goal is to compare NAb responses to different AAV serotypes. The usage of animal models may provide extra insights into studying the NAb response to AAVs and expand the methods used to determine the level of these NAbs.

A study by Mingozzi et al. (2018) demonstrated that, despite promising outcomes in animal models, human trials for AAV gene therapy have encountered unexpected immune reactions [60]. These differences highlight a challenge in translating findings on immune responses to AAVs from animal models to humans [61].

Mice and non-human primates (NHPs) are the most widespread animal models that are used to study the immune response to AAVs. However, the composition and role of immune cells differ between humans and mice [62]. Mestas and Hughes (2004) highlighted that human immune systems contain unique immune cell subsets not found in mice (CXCR1, CD58 (LFA-3), CD40 on ECs, etc.), which can affect the study of viral infections and the immune response to AAV vectors [62]. NHP models have demonstrated the potential for unexpected immune reactions, including the activation of memory T cells and antibody responses that were not fully predicted by rodent models [63]. Despite their advantages, NHPs are not without limitations [64]. Rhesus macaques display a higher number of certain immune cells, such as CD4^+^/CD8^+^ double-positive T cells, than humans [65]. They may react differently to AAV vectors compared to cynomolgus macaques, leading to variability in study outcomes [64,66,67]. Furthermore, in vaccine studies, while NHPs such as macaques often develop strong NAb responses, their immune system does not always mimic human long-term immunity or antibody decay rates, as seen in COVID-19 vaccine research [68]. Thus, there is a need to develop more appropriate systems to test human or human-like immune responses to AAVs.

Humanized mouse models would be valuable in studying the immune response to AAVs. These models aim to address the limitations of traditional animal models, offering more accurate predictions of human immune responses. Studies using these mice have shown that, while humanized models can replicate some aspects of the human immune response, such as T-cell activation and antibody production, they may not fully recapitulate the complexity of human immune reactions to viral infections, particularly those involving regulatory T cells and innate immune cells such as plasmacytoid dendritic cells (pDCs) [69].

A breakthrough mouse model with a complete, functional human immune system and human-like gut microbiome was created by the Paolo Casali group [70]. The most interesting result demonstrated in this model, in terms of detecting the human humoral response, is that these (truly humanized) THX mice showed mature neutralizing antibody responses to Salmonella Typhimurium and the SARS-CoV-2 virus after vaccination with Salmonella flagellin and the Pfizer COVID-19 mRNA vaccine, respectively. It would be desirable to test NAb formation, which will reflect the human humoral response, in these mice to other viruses, including AAVs.

Knock-in mice engineered to express human immune components offer a controlled system for studying AAV interactions. For instance, different studies used knock-in mice with human immunoglobulin loci, providing further insight into human-specific immune responses [71,72,73].

To summarize, the previously described animal models reflect human immune responses and develop humoral responses to AAVs after vector injection to varying extents. However, the induced humoral response to AAVs is usually measured by in vitro assays, the limitations of which were also discussed above. Next, we describe animal models that allow the measurement of human-like NAb responses, fully avoiding in vitro neutralization tests.

This approach involves inducing a human-like humoral immune system in animals, followed by AAV injection. The effectiveness of vector neutralization is compared to the same line of mice that were not humanized.

In a study by Lan Sun et al. [74], researchers generated a mouse model of passive immunity to quantitatively assess anti-AAV8 NAb titers. C57BL/6 mice were first injected with rhesus macaque sera followed by scAAV2/8.CB.hAAT. The level of anti-hAAT Abs was measured using ELISA and inversely corresponded, according to the authors’ study design, to the level of anti-AAV NAbs. This study showed that this in vivo NAb assay was more sensitive than an in vitro NAb assay; serum samples with low titers (<1:5 NAbs) were detected as positive to the anti-AAV8 response only by the in vivo assay. Lili Wang et al. introduced this approach, but they transferred monkey sera to C57BL/6 mice prior to the injection of AAVs with cFIX, as in [75]. cFIX expression levels in mouse plasma were indicators of AAV neutralization.

Thus, in the first example, the level of NAbs to AAVs was evaluated relative to the anti-transgene antibody level, whereas in the second example, it was relative to transgene expression. The level of viral DNA and/or transgene RNA may be included in the study design when this approach is used.

To summarize, animal models may be used in two ways. The first one is to use animals with a modified immune system (humanized animal models). The second way is to use animals with an immune system close to that of humans, inject them with the AAV, and measure NAbs using in vitro tests. In other words, the last method may be used without the necessity for in vitro neutralization tests, which expands the possibilities when it comes to the detection of AAV NAbs.

## 6. Ex Situ Assays with Whole Perfused Explants

The whole human explant offers another valuable perspective for evaluating the level of NAbs to AAVs. In this context, Marti Cabanes-Creus et al. (2024) introduced an ex situ system of normothermic human liver explants. One of them was perfused with human blood in the presence of anti-AAV NAbs that contained an AAV library [76]. The other liver explant was perfused with the AAV library only. The level of AAV neutralization corresponded to differences between two explants in the level of viral DNA and transgene cDNA, as determined by NGS. This method did not require in vitro neutralization assays.

This model is characterized by the ability to preserve the structure of human tissue, deliver the drug through a natural network of capillaries, and observe the neutralization of AAVs in the presence of resident macrophages and Kupffer cells. This allows plasma to be passed through the perfusion system under conditions close to physiological ones. The authors showed that in the presence of human plasma containing anti-AAV NAbs, vectors derived from AAV2/AAV3b were extensively neutralized, unlike AAV8-derived variants. The authors noticed that AAVs were rapidly cleared from the perfusate if the explant contained NAbs to the serotype. This may have been mediated by Kupffer cells and could not be represented in vitro.

In general, the use of ex situ normothermic perfusion of human organs opens up prospects for studying the neutralization of AAVs in an environment containing different tissues and cell types, including the resident immune cells of the organ. Besides the liver, models of isolated hearts perfused with AAVs already exist [77,78] and may potentially be used for detecting neutralizing antibody activity if human plasma is added to the perfusing solution.

Ex situ perfusion of human organs with NAbs and AAVs has several limitations. The availability of donor material is generally limited and further restricted by exclusion criteria for transplantation. The maintenance of the explants in a viable state for several days is also a difficult methodological task. Upon connection to the perfusion unit, donor organs may undergo reperfusion damage and fragmentation, compromising the capillary structure and, subsequently, the virus’ distribution pathway. It is shown that, after six days of perfusion, markers of necrosis in the liver explant increased and organ functionality declined [79]. Moreover, the normothermic perfusion explant model is limited by the absence of circulating immune cells, which may potentially be involved in the AAV response. The advantages of this model include that it does not require in vitro neutralization tests and partially reflects the interaction of AAVs with cells with immune function as it occurs in the whole organism.

## 7. Antigenic Map of AAVs Created on Published Data

The surface capsid proteins of different variants (strains, serotypes, and isolates) of the same virus always have a certain homology in sequence and structure [47,80,81]. Such homology, which is closely linked with evolutionary relationships, may result in the similarity of the physicochemical properties of the protein surface. This leads to cross-reactivity, when antibodies formed by the host in response to one variant of the virus neutralize another variant.

Despite several attempts to predict cross-reactivity in silico [82,83,84], the gold standard of antigenic similarity estimation is the mass assessment of neutralizing properties of diverse serum samples. The serum samples can be taken from model animals immunized with a specific variant. However, as previously discussed, animals can develop similar, but not identical, humoral responses to humans, which leads to unpredictable deleterious immune responses in AAV-based GT clinical trials. Sera from conditionally healthy (not infected) individuals or those who have been infected by a known virus serotype are preferable [85], but in the case of AAVs, this is not achievable. Titer neutralization data obtained from individuals who have been vaccinated with an AAV GT drug based on a known serotype provide sufficient data about the formation of cross-reactive antibodies.

Animal or human serum neutralization capacities are usually tested in vitro, the advantages and limitations of which were discussed above. If these limitations are overcome or compromises are found, the reliable comparison of humoral responses to different AAV serotypes becomes possible. This is highly preferred in studies in which, for example, chimeric AAVs are developed with the purpose of evading pre-existing humoral responses [49,86,87,88].

Over the past decades, many different studies have been conducted that have assessed the prevalence of neutralizing antibodies against various AAV serotypes in diverse human populations [4,7,89]. Such investigations not only provide critical information about the percentage of seropositive individuals (and hence the potential for the clinical use of a particular virus variant) but also allow the assessment of antigenic similarities between different AAV serotypes. However, these data are often segmental and difficult to visualize and analyze.

Previously, this problem appeared with cross-reactive immune responses to influenza virus strains or SARS-CoV-2 variants, and was solved by the antigenic cartography approach developed by Dereck [90,91,92,93,94,95]. This approach visualizes a large dataset of titers for several serotypes on a map. The closer the serotypes are located to each other on the antigenic maps (represented by areas), the more antigenically similar they are. Sera from various patients, represented by circles, are closer to the variants that they neutralize more. One grid line on the map corresponds to a twofold dilution in the virus neutralization assay and is called an “antigenic unit”.

Here, we collected data from four publications in which neutralization titers of sera were performed [59,96,97,98]. We considered all published datasets containing patient-level human titer data (not the percentage of neutralization as in [98]). In many studies, raw titer data are not provided, and percentages of seroprevalence are available [99]. Such studies were not included in the analysis. At least three serotypes tested in this study were necessary to include in the dataset in the antigenic map construction. The description of cohorts and AAV serotypes for which neutralization titers are performed in each of these studies is summarized in Table 2.

**Figure 4:**
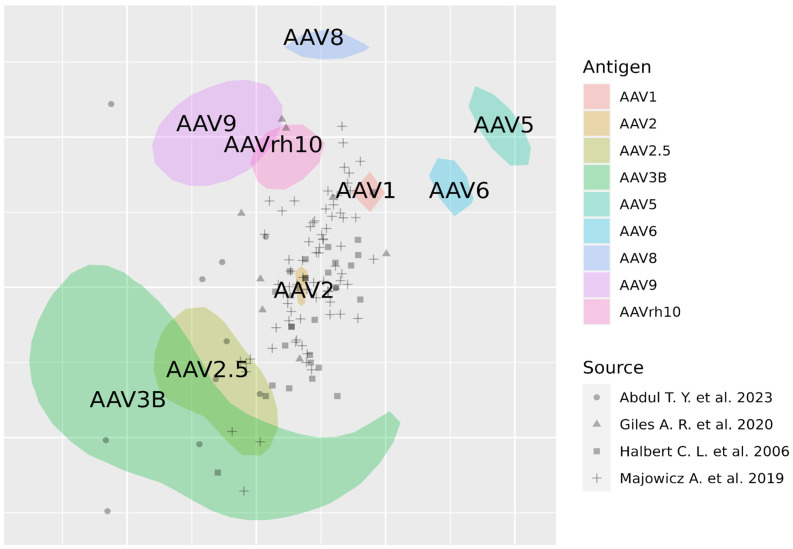
Antigenic map of adeno-associated viruses (AAVs) constructed on published titer neutralization data. AAV serotypes are represented by areas, and sera by circles. Areas considered experimental and the statistical uncertainty of their location. One grid line corresponds to one antigenic unit [59,96,97,98].

Titer data were analyzed using the Racmacs R package [100] and plotted on an antigenic map (Figure 4). Experimental areas and the statistical uncertainty of their location were determined by both pairwise similarity (ability to neutralize the same antigens) and their proximity to related antigens.

The map, in general, represents some expected patterns. For example, AAV2 is located in the center of the cloud of sera samples as the variant with the most common neutralization in the human population. AAV5 and AAV2 are far away from each other in the antigenic map. It is known that these two variants have low sequence homology [101]. Here, we show that they may also be antigenically distinct. Surprisingly, the serotypes AAV9 and AAVrh10 turn out to be closely related, despite being more phylogenetically similar to AAV8 [102]. AAV1 and AAV6, despite very high sequential and structural homology, demonstrate distinct response patterns. These observations may serve as a basis for further validation and more in-depth research.

It should be noted that the represented antigenic map has several limitations. Firstly, the used titers were obtained in different conditions in different laboratories, leading to different titer ranges for the same serotype in different studies. Titer data from included studies are heterogeneous because different serotypes were tested. The ability to neutralize AAV2 was tested in all presented studies, so the position of this serotype on the map can be considered the most precise compared with serotypes that were used in only one study. The number of studies we included in our analysis is low due to the limited availability of the raw data on neutralization titers in publications. Together, these limitations can lead to the positions of antigens on the map being inaccurate. Thus, AAV3B has a wide area because of the statistical uncertainty of its location. In addition, an insufficient number of cross-reactive serum samples located between serotypes on the map can be determined. In a study by Temilola Abdul et al., individuals with autoimmune disease (osteoarthritis) were included, although their humoral response may differ from healthy donors [97].

Taken together, for the robust and reliable determination of the antigenic relationship between AAV serotypes, a systematic study is needed. This study must include many AAV variants (ideally, all variants of clinical importance) and a large homogeneous cohort of healthy individuals that are tested simultaneously. The limitations of neutralization assays should be reduced to a minimum using the recommendations that we have previously discussed. Such a study would provide valuable information for subsequent capsid designs, enabling them to evade neutralization by pre-existing antibodies. Despite significant community efforts during recent years, overcoming pre-existing humoral immune responses remains a difficult challenge. An antigenic map serves as a representative approximation of the antigenic relationships of AAV serotypes, which may drive the optimal selection of AAV serotypes for patients who are seropositive for some AAV variants.

## 8. Prospects and Future Directions

Pre-existing humoral immune responses to AAVs are a well-known limitation of GT applications. The measurement of AAV NAb titers and the determination of the threshold level acceptable in GT are still being discussed. Here, we have shown the differences in antibody titers that are exclusion criteria for GT trials. They may be explained by the absence of standardization of the assays used for the determination of specific anti-AAV Abs. We focused on the details of in vitro assays in the context of AAVs and have shown that the quality of VPs (ratio of full/empty capsids) influences the number of transduced target cells and, as a result, the NAb titers, so this parameter should be under consideration in in vitro tests. Additionally, an equal ratio of different serotype VPs and serum volumes should be used to obtain relevant titers. To overcome the problem of the different transduction efficiency of serotypes, we introduced the recommendation to transfer such volumes of serum and VP mixtures to cells, which, in the absence of serum, leads to the necessary percentage of transduction of target cells. Alternative approaches to the assessment of NAb titers, such as using animal models and perfused organs, were reviewed. These methods provide more physiological systems and can potentially overcome the differences in the transduction ability of AAV serotypes. A more precise description of the methods used in the assessment of anti-AAV NAb titers and following the good practice recommendations that we introduced here are necessary to increase the relevance and homogeneity of studies. The reliable comparison of titers to different AAV serotypes could help researchers design more effective vectors that avoid pre-existing immunity. The antigenic mapping approach is suitable for visualizing antigenic relationships between AAV serotypes and revealing cross-reactive serum samples. However, more reliable titer data are required to construct a more precise and sustainable antigenic map.

## Figures and Tables

**Table 1:** Selected examples of over 30 clinical studies employing AAVs.

Disease	Status	Phase	Exclusion Criteria and Method of Detection of Anti-AAV Abs	Administration	Sponsor	ClinicalTrials.gov ID
**AAV9**						
Muscular Atrophy Type 1	COMPLETED	Phase 1	Binding antibody titers > 1:50, ELISA	Intravenous	Novartis (Novartis Gene Therapies) Basel, Switzerland	NCT02122952
Mucopolysaccharidosis (MPS) IIIA	RECRUITING	Phase 2 Phase 3	Binding antibody titers ≥ 1:100, ELISA	Intravenous	Ultragenyx Pharmaceutical Inc Novato, CA, USA	NCT02716246
Mucopolysaccharidosis (MPS) IIIA	TERMINATED	Phase 1 Phase 2	Binding antibody titers ≥ 1:100, ELISA	Intravenous	Abeona Therapeutics, Inc Cleveland, OH, USA	NCT03315182
Late Infantile Neuronal Ceroid Lipofuscinosis 6 (vLINCL6)	COMPLETED	Phase 1 Phase 2	Binding antibody titers > 1:50, ELISA	Intrathecally into the lumbar spinal cord region	Amicus Therapeutics Philadelphia, PA, USA	NCT02725580
**AAVrh10**						
Late Infantile Krabbe Disease Treated Previously With HSCT (REKLAIM)	RECRUITING	Phase 1 Phase 2	Binding antibody titers > 1:100, ELISA (these criteria will not apply to children screened before they have received HSCT or for children who sign the informed consent within 60 days of HSCT)	Intravenous	Forge Biologics, Inc Grove City, OH, USA	NCT05739643
Alpha-1 Antitrypsin (A1AT)	COMPLETED	Phase 1 Phase 2	Neutralizing antibody titer ≥ 1:5, neutralizing Ab	Intravenous or intrapleural	Adverum Biotechnologies, Inc. Redwood City, CA, USA	NCT02168686
Hemophilia B	TERMINATED	Phase 1 Phase 2	Neutralizing antibody titer > 1:5, neutralizing Ab	Intravenous	Ultragenyx Pharmaceutical Inc Novato, CA, USA	NCT02618915
**AAV2**						
Advanced Parkinson’s Disease	COMPLETED	Phase 1	Total antibody titer > 1000, ELISA	Bilateral Stereotactic Convection—Enhanced Delivery	National Institute of Neurological Disorders and Stroke (NINDS) Bethesda, MD, USA	NCT01621581
Aromatic L-amino Acid Decarboxylase (AADC) Deficiency	COMPLETED	Phase 2	Patients with a neutralizing antibody titer over 1200-fold or an ELISA OD over 1 cannot be recruited into this trial.	Intracerebral	National Taiwan University Hospital Taipei, Taiwan	NCT02926066
Leber congenital amaurosis (LCA)	ACTIVE, NOT RECRUITING	Phase 1	AAV antibody titers greater than two standard deviations above normal at baseline	Subretinal	University of Pennsylvania Philadelphia, PA, USA	NCT00481546
Hemophilia B	TERMINATED	Phase 1	Presence of neutralizing antibodies, AAV2/6 vector	Intravenous	Sangamo Therapeutics Richmond, CA, USA	NCT02695160
**AAV8**						
Hemophilia B	TERMINATED	Phase 1	Neutralizing antibody titer > 1:5	Intravenous	Spark Therapeutics, Inc. Philadelphia, PA, USA	NCT01620801
Late-Onset Pompe Disease (FORTIS)	RECRUITING	Phase 1 Phase 2	Neutralizing antibody titer > 1:20	Intravenous	Astellas Gene Therapies Sanford, NC, USA	NCT04174105
Homozygous Familial Hypercholesterolemia (HoFH)	TERMINATED	Phase 1 Phase 2	Neutralizing antibody titer > 1:10	Intravenous	REGENXBIO Inc Rockville, MD, USA	NCT02651675
Hemophilia B	TERMINATED	Phase 1 Phase 2	Neutralizing antibody titers ≥ 1:5	Intravenous	Baxalta, now part of Shire Bannockburn, IL, USA	NCT04394286
Hemophilia B	ACTIVE, NOT RECRUITING	Phase 1	Detectable antibodies reactive with AAV8	Intravenous	St. Jude Children’s Research Hospital Memphis, TN, USA	NCT00979238
cocaine use disorder	RECRUITING	Phase 1	Patients with detectable pre-existing immunity to the AAV8 capsid as measured by AAV8 transduction inhibition and AAV8 total antibodies.	Intravenous	W. Michael Hooten Rochester, MN, USA	NCT04884594
The Human Immunodeficiency Virus (HIV)	ACTIVE, NOT RECRUITING	Phase 1	Titer of pre-existing antibodies to capsid > 1:90	Intramuscular	National Institute of Allergy and Infectious Diseases (NIAID) North Bethesda, MD, USA	NCT03374202
Hemophilia B	TERMINATED	Phase 1	Neutralizing antibody titer > 1:5	Intravenous	Spark Therapeutics, Inc. Philadelphia, PA, USA	NCT01620801
**AAVrh74**						
Limb–Girdle Muscular Dystrophy, Type 2D (LGMD2D)	COMPLETED	Phase 1 Phase 2	Binding antibody titers ≥ 1:50, ELISA	Isolated limb infusion (ILI)	Sarepta Therapeutics, Inc. Cambridge, MA, US	NCT01976091
Dysferlinopathies	COMPLETED	Phase 1	Binding antibody titers > 1:50, ELISA	Extensor digitorum brevis (EDB) muscle	Sarepta Therapeutics, Inc. Cambridge, MA, USA	NCT02710500
Duchenne Muscular Dystrophy	COMPLETED	Phase 1	Binding antibody titers ≥ 1:50, ELISA	Extensor Digitorum Brevis (EDB) muscle	Jerry R. Mendell Columbus, OH, USA	NCT02376816
**AAV5**						
Hemophilia A	TERMINATED	Phase 1 Phase 2	Absence of pre-existing antibodies against the AAV5 vector capsid, measured by total AAV5 antibody ELISA	Intravenous	BioMarin Pharmaceutical San Rafael, CA, USA	NCT03520712
Hemophilia B	COMPLETED	Phase 1 Phase 2	Neutralizing antibodies against AAV5 at Visit 1	Intravenous	CSL Behring King of Prussia, PA, USA	NCT02396342
Arthritis	UNKNOWN STATUS	Phase 1	Presence of neutralizing antibody (NAb) titers against adeno-associated virus type 5 (AAV5) and/or hIFN-β.	Intra-articular	Arthrogen Amsterdam, Netherlands	NCT02727764
Haemophilia A	COMPLETED	Phase 1 Phase 2	Detectable pre-existing immunity to the AAV5 capsid as measured by AAV5 transduction inhibition or AAV5 total antibodies	Intravenous	BioMarin Pharmaceutical San Rafael, CA, USA	NCT02576795
**AAV1**						
CMT1A	SUSPENDED Vector has not been produced	Phase 1 Phase 2	Binding antibody titers ≥ 1:50, ELISA	Intramuscular	Nationwide Children’s Hospital Columbus, OH, US	NCT03520751
Duchenne Muscular Dystrophy	COMPLETED	Phase 1 Phase 2	Binding antibody titers > 1:50, ELISA	Intramuscular	Jerry R. Mendell Columbus, OH, USA	NCT02354781
Heart Failure	TERMINATED	Phase 2	Neutralizing antibody titers ≥ 1:2	Intracoronary	Assistance Publique—Hôpitaux de Paris Paris, France	NCT01966887
Advanced Heart Failure	COMPLETED	Phase 2	Neutralizing antibody titers ≥ 1:2	Intracoronary	Celladon Corporation San Diego, CA, USA	NCT01643330
Limb–Girdle Muscular Dystrophy Type 2C	COMPLETED	Phase 1	Pre-injection neutralizing anti-AAV1 antibodies titer superior or equal to 1/800.	Intramuscular	Genethon Evry, France	NCT01344798
Becker Muscular Dystrophy and Sporadic Inclusion Body	COMPLETED	Phase 1	Neutralizing antibody titers ≥ 1:1600, ELISA	Intramuscular	Nationwide Children’s Hospital Columbus, OH, USA	NCT01519349
Heart Failure	RECRUITING	Phase 1	Anti-AAV1 neutralizing antibodies	Antegrade epicardial coronary artery infusion	Sardocor Boston, MA, USA	NCT06061549
**Others**						
Hemophilia A	ACTIVE, NOT RECRUITING	Phase 3	Anti-AAV6 neutralizing antibodies	Intravenous	Pfizer New York, NY, USA	NCT04370054
Hemophilia B	COMPLETED	Phase 2	Neutralizing antibodies reactive with AAV, Spark100 above and/or below a defined titer	Intravenous	Pfizer New York, NY, USA	NCT02484092
Hemophilia A	ACTIVE, NOT RECRUITING	Phase 1 Phase 2	Detectable antibodies reactive with the AAVhu37capsid.	Intravenous	Bayer Leverkusen, Germany	NCT03588299
Hemophilia A	COMPLETED	Phase 1 Phase 2	Detectable antibodies reactive with the AAV-Spark200 capsid	Intravenous	Spark Therapeutics, Inc. Philadelphia, PA, USA	NCT03003533
Hemophilia A	COMPLETED	Phase 1 Phase 2	Detectable antibodies reactive with the AAV-Spark capsid	Intravenous	Spark Therapeutics, Inc. Philadelphia, PA, USA	NCT03734588
Fabry Disease	ACTIVE, NOT RECRUITING	Phase 1 Phase 2	Presence of high-titer neutralizing antibodies to the 4D-310 capsid, or presence of a high-antibody titer to AGA	Intravenous	4D Molecular Therapeutics Emeryville, CA, USA	NCT04519749

**Table 2:** Summary of data used to construct the antigenic map.

Group	Cohort Description	In Vitro Neutralization Test Features	Reference	AAV Serotypes Used								
				AAV1	AAV2	AAV2.5	AAV3B	AAV5	AAV6	AAV8	AAV9	AAVrh10
1	Osteoarthritis patient population	Features include 7.5 × 10^6^ transducing units of AAV-GFP + 56 µL diluted synovial fluid dilution, 5 × 10^4^ HIG-82 cells/well, and GFP expression analyzed by FACS. The NAb titer is given as the dilution of synovial fluid required to obtain 50% inhibition of transduction by AAV.GFP, as compared to cells incubated with AAV.GFP alone.	Abdul T. Y. et al., 2023 [97]		☑	☑		☑				
2	Normal human donors	Features include 10^9^ AAV-LacZ vg/well + 2-fold serial diluted serum samples (initial dilution, 1:20), 10^5^ Huh7 cells/well, and luciferase activity as measured by a microplate luminometer. The NAb titer was reported as the highest serum dilution that inhibited AAV transduction by 50%, compared with the mouse serum control.	Giles A. R. et al., 2020 [98]		☑		☑			☑	☑	☑
3	Healthy and hemophilia B patient populations	Features include 70 ul of virus + serial diluted serum samples (initial dilution, 1:20), HEK293T cells, and luciferase activity as measured by a microplate luminometer. NAb titer (IC_50_) is the dilution at which antibodies inhibit HEK293T cell transduction with AAV-luc by 50%.	Majowicz A. et al., 2019 [96]	☑	☑			☑	☑	☑		
4	Healthy and cystic fibrosis patient populations	Features include 10^8^ AAV-AP vg/well + 100 ul diluted serum samples (initial dilution, 1:20), HTX cells, and AP-positive focusforming units were measured. The highest dilution of serum that inhibited AAV transduction by 50% or more compared with the untreated vector was defined as the neutralizing titer.	Halbert C. L. et al., 2006 [59]		☑			☑	☑			

A tick mark in the table indicates the described neutralizing antibody titers for the AAV serotype.
